# Evaluation of cattle sub-species and growth-promoting technology on growth performance, carcass characteristics, and enteric gas flux of steers finished in winter feedlot conditions

**DOI:** 10.1093/jas/skaf448

**Published:** 2025-12-29

**Authors:** Ashley K Schilling-Hazlett, Kimberly R Stackhouse-Lawson, Tony C Bryant, Sara E Place, John P Ritten, Juan J Vargas, Irene A Reis, Edilane C Martins, Maya A Swenson, Erin N Burke, Rhyse K Campion, Cesar Velasquez, Anna M Shadbolt, Pedro H V Carvalho

**Affiliations:** CSU AgNext, Department of Animal Sciences, Colorado State University, Fort Collins, CO 80523; CSU AgNext, Department of Animal Sciences, Colorado State University, Fort Collins, CO 80523; Five Rivers Cattle Feeding, Johnstown, CO 80534; CSU AgNext, Department of Animal Sciences, Colorado State University, Fort Collins, CO 80523; CSU AgNext, Department of Animal Sciences, Colorado State University, Fort Collins, CO 80523; CSU AgNext, Department of Animal Sciences, Colorado State University, Fort Collins, CO 80523; CSU AgNext, Department of Animal Sciences, Colorado State University, Fort Collins, CO 80523; CSU AgNext, Department of Animal Sciences, Colorado State University, Fort Collins, CO 80523; CSU AgNext, Department of Animal Sciences, Colorado State University, Fort Collins, CO 80523; CSU AgNext, Department of Animal Sciences, Colorado State University, Fort Collins, CO 80523; CSU AgNext, Department of Animal Sciences, Colorado State University, Fort Collins, CO 80523; CSU AgNext, Department of Animal Sciences, Colorado State University, Fort Collins, CO 80523; CSU AgNext, Department of Animal Sciences, Colorado State University, Fort Collins, CO 80523; CSU AgNext, Department of Animal Sciences, Colorado State University, Fort Collins, CO 80523

**Keywords:** *Bos taurus indicus*, *Bos taurus taurus*, growth performance, growth-promoting technology, methane gas flux, sustainability

## Abstract

The study objectives were to evaluate the comparative growth performance, carcass characteristics, and gas flux of yearling *Bos taurus indicus* (BI; Brahman) and *Bos taurus taurus* (BT; Angus) steers managed with (GPT+) and without (GPT−) the use of growth-promoting technology (GPT) in winter conditions. One hundred BI (initial body weight [IBW] = 364 ± 22 kg) and 100 BT (IBW = 323 ± 17 kg) steers were fed for 180 d in two consecutive phases. In Phase 1, day 0–83, steers of each sub-species were blocked by IBW and randomly assigned to a treatment (10 hd/pen, 5 pens/treatment). In Phase 2, day 84–180, steers were moved to Climate Smart Research Pens, where each treatment was randomly assigned to a research pen equipped to measure individual feed intake and gas flux (50 hd/pen, 1 pen/treatment). Data were analyzed with R (R Core Team, 2021, v. 4.4.1) software to assess the fixed effects of cattle sub-species, treatment, and the sub-species × treatment interaction. In Phase 1, IBW differed (*P *< 0.01) by sub-species, but did not differ (*P *= 0.98) by treatment. Dry matter intake (DMI), average daily gain (ADG), and feed efficiency (G:F) were greater (*P *< 0.01) for BT, resulting in greater final body weight (FBW) for Phase 1. Within sub-species, DMI, on a total daily basis and as a percentage of body weight, did not differ (*P *≥ 0.09) between treatments in Phase 1, but ADG, G:F, and FBW were greater (*P *≤ 0.04) for GPT+ than GPT−. In Phase 2, G:F and DMI when represented on a total daily and as a percentage of BW basis had a sub-species × treatment interaction (*P *≤ 0.04) where greater increases in G:F and DMI were observed between GPT+ and GPT− for BI than for BT. Steers managed with GPT and BT steers had greater (*P *≤ 0.01) ADG and FBW. Calculated yield grade and backfat thickness were greater (*P *< 0.01) for BT steers, and BT had more (*P *< 0.01) USDA Choice and Prime quality grades than BI. Longissimus muscle area was greater (*P *< 0.01) for BT and GPT+ steers. Daily methane (CH_4_) emitted was less for BI steers; however, CH_4_ as a proportion of DMI and gross energy intake were less (*P *< 0.01) for BT steers. There was a sub-species × treatment interaction (*P *< 0.01) for CH_4_ per unit ADG, where a decrease was observed between GPT− and GPT+ for BI, while no difference was observed for BT. In conclusion, BT had greater growth performance and carcass quality, but observations differed by cattle sub-species when CH_4_ was reported on an absolute versus yield or intensity basis.

## Teaser

In the beef industry, the use of growth-promoting technology and cattle sub-species selection are examples of proposed innovations to improve the adaptive capacity and resiliency of the beef supply chain to ensure food security for current and future generations. In the present study, *Bos taurus taurus* steers consumed greater quantities of feed with greater efficiency, resulting in heavier final body and carcass weight, as well as yielded more USDA Choice and Prime carcasses compared to *Bos taurus indicus* cattle that emitted less methane per day.

## Introduction

As climate change progresses, there is a growing need to emphasize both climate mitigation and adaptation to combat increasingly severe, interconnected, and often irreversible climate impacts that disrupt supply chains (Pörtner et al., 2022). Beef cattle production systems are simultaneously vulnerable to the impacts of climate change and contributors to the progression. In the United States (US), enteric methane (CH_4_) emissions are the largest source of emissions from beef cattle and comprise approximately 2% of total US greenhouse gas (GHG) emissions (EPA, 2024). Conversely, an increasing concern among US beef producers is the impact that a changing climate can have on animal performance and economic well-being, threatening the long-term viability of the beef industry (Capper and Hayes, 2012; Rojas-Downing et al., 2017). To address plausible opportunities for mitigation and adaptation in the beef supply chain, cattle selection (Reichhardt et al., 2023) and managing beef cattle with growth-promoting technology (GPT; Capper and Hayes, 2012) have been proposed.

Beef cattle production in the US occurs across a large range of climatic zones from subtropical to temperate. To address increasingly variable and extreme thermal conditions, researchers have investigated the potential benefits of increasing the proportion of *Bos taurus indicus* (BI) cattle in the US cattle herd. In the US, BI cattle were first introduced to the Gulf Coast region to improve adaptability of beef cattle to the subtropical environmental conditions (Cartwright, 1980; Huffman et al., 1990). The greatest advantage of BI cattle in the US has been realized in crossbreeding programs, where heterosis contributes to improved production characteristics while enhancing tolerance to heat, disease, and parasites, particularly in the southern and southeastern US, where cattle are exposed to hot, humid climates and poor quality forages (Paschal et al., 1995). Most BI cattle in the US were imported from Brazil, although their ancestral origin is India, in contrast to *Bos taurus taurus* (BT) cattle which are of European origin (Sanders, 1980). Morphological traits that distinguish BI from BT cattle include the cervico-thoracic hump, large pendulous ears, and excessive skin along the ventral body surface, chest, and neck (Utsunomiya et al., 2019). Beyond physical characteristics, BI and BT cattle differ in several important biological and production characteristics, including (1) heat and cold tolerance, (2) reproduction, (3) growth and maturation rates, (4) temperament, and (5) complementarity in crossbreeding systems (Cartwright, 1980). As a result of these differences, economic (i.e., growth performance and carcass quality) and social concerns (i.e., consumer acceptance of meat quality and cattle temperament) have been documented for BI cattle (Boyles and Riley, 1991; Voisinet et al., 1997; Wright et al., 2018).

Still, an eminent need exists to identify adaptive strategies for beef cattle production systems as most regions are projected to experience increased frequency and severity of extreme heat events relative to the 1950s and decreased frequency and severity of cold extremes (IPCC, 2021). Already, migratory shifts in the number of cattle and beef operations are apparent with cattle inventory trends reporting cattle moving northward to the central part of the US as well as into more concentrated operations (Fancher et al., 2025). Before recommendations to increase the proportion of BI cattle are explored, it is important to garner more information regarding their feedlot growth performance and carcass characteristics in more temperate regions (Cartwright, 1980). Additionally, improved growth performance, which can be achieved via the use of GPT, is suggested to be one of the most effective mitigation strategies to decrease GHG emissions from beef production per unit of feed consumed and product produced (Boadi et al., 2004; Monteny et al., 2006; Capper and Hayes, 2012; Stackhouse et al., 2012). Despite potential productivity and efficiency improvements resulting from the use of GPT, previous research has reported that different cattle breeds may respond differently to GPT (Reichhardt et al., 2023). Therefore, it is important to quantify differences in the effect of GPT to best inform future cattle selection and management decisions.

Further, it is important to quantify individual gas flux measurements from BI and BT cattle managed with and without the use of GPT in a temperate geographic region representative of where the US cattle herd is currently concentrated, to understand the impact of GHG emissions from the fed cattle sector. The objectives of the present study were to evaluate enteric gas flux of CH_4_, carbon dioxide (CO_2_), oxygen (O_2_), and hydrogen (H_2_), growth performance, and carcass characteristics of BI and BT sub-species of cattle managed with and without the use of anabolic implants, an in-feed antibiotic, ionophore, and beta-adrenergic agonist in a temperate climate in winter feedlot conditions. The authors hypothesized that BT steers and steers managed with GPT would exhibit greater growth performance, primarily due to greater feed intake and feed efficiency compared to BI steers and steers not managed with GPT. Predicted differences in growth performance were hypothesized to influence enteric gas flux emissions, where cattle that consumed more feed were expected to exhibit greater CH_4_ emissions; thus, BT steers were expected to produce more CH_4_ than BI steers.

## Materials and Methods

This experiment was conducted at the Colorado State University Agricultural Research, Education, and Development Center experiment station in Fort Collins, CO, from October 2023 to April 2024, consisting of a 180 d feeding period divided into two consecutive phases. All procedures involving animals were approved by the Colorado State University Institutional Animal Care and Use Committee (protocol no. 3712). All animals evaluated in the present study were sourced in two lots by a commercial cattle feeder, where lot one was comprised of BT steers from Montana and lot two of BI steers sourced from Texas. All steers were allotted 38 d to acclimate to the research facility prior to study initiation.

### Environmental conditions

Environmental variables were collected using the publicly available CoAgMET service to acquire ambient air temperature (°C; T_a_), relative humidity (%, RH), solar radiation (W/m^2^, SR), precipitation (mm, P), and wind speed (WS, m/s). Data were collected continuously throughout the experimental period from an on-site weather station (station no. ftc03) located 9.7 km northeast of Fort Collins at an elevation of 1557.5 m at the Colorado State University Agricultural Research, Education, and Development Center (40.65 °N, −105 °E).

Weather station data collected during the 180 d feeding period is presented in Table 1. An average *T*_a_ of 2.2 °C was exhibited, with a minimum of −30.2°C and a maximum of 28.2°C. January was the coldest month, with an average *T*_a_ of −4.2 °C, exhibiting a minimum of −30.2°C and a maximum of 16°C. Average RH during the feeding period was 56.8%, with a minimum of 7.9% and an average maximum of 98.7% during the experimental period. Moreover, average SR, WS, and P were 135.6 W/m^2^, 2.5 m/s, and 0.02 mm/month, respectively, during the experimental period.

**Table 1.** Descriptive statistics of environmental variables measured during the 180 d feeding period

	Environmental variable^1^														
	T_a_			RH			SR			WS			P		
Month^2^	Min^2^	Max^2^	Mean	Min^2^	Max^2^	Mean	Min^2^	Max^2^	Mean	Min^2^	Max^2^	Mean	Min^2^	Max^2^	Mean
**OCT**	−11.5	28.2	5.3	8.5	96.7	62.3	0.0	682.7	130.4	0.1	3.4	1.6	0.00	1.02	0.01
**NOV**	−14.0	23.2	3.2	10.7	94.9	54.6	0.0	569.3	113.6	0.2	7.4	2.1	0.00	0.51	0.00
**DEC**	−10.4	17.6	1.2	11.5	98.6	56.8	0.0	549.5	83.3	0.1	11.5	2.7	0.00	1.52	0.01
**JAN**	−30.2	16.0	−4.2	15.5	94.5	61.3	0.0	559.8	102.8	0.1	10.9	2.3	0.00	1.78	0.00
**FEB**	−16.2	16.9	1.8	9.2	98.7	59.3	0.0	727.8	142.4	0.2	12.4	2.6	0.00	4.32	0.04
**MAR**	−9.7	18.8	3.7	7.9	97.5	58.9	0.0	876.8	181.4	0.1	11.1	2.6	0.00	2.03	0.03
**APR**	−4.3	26.3	9.8	8.3	93.0	40.9	0.0	942.3	237.9	0.4	15.4	4.0	0.00	2.54	0.01

1 T_a_, ambient air temperature, °C; RH, relative humidity, %; SR, solar radiation, W/m^2^; WS, wind speed, m/s; P, precipitation, mm/month.

2 Month of 180-d feeding period were OCT, October; NOV, November; DEC, December; JAN, January; FEB, February; MAR, March; and APR, April

3 Min, minimum; Max, maximum.

### Animals and experimental design

Animals consisted of 100 BI (Brahman) yearling steers (initial body weight [IBW] = 364 ± 22 kg) and 100 BT (Angus) yearling steers (IBW = 323 ± 17 kg). Steers of each sub-species were randomly assigned to a management treatment, managed with GPT (GPT+) or without GPT (GPT−), in a 2 × 2 factorial arrangement. Steers in GPT+ received an anabolic implant on 0 d (100 mg trenbolone acetate/14 mg estradiol benzoate; Synovex Choice, Zoetis, Parsippany, NJ, USA) and 84 d (200 mg trenbolone acetate/28 mg estradiol benzoate; Synovex Plus, Zoetis). Steers in GPT+ also received an in-feed ionophore (35 g/ton on dry matter [DM] basis; monensin, Rumensin, Elanco, Greenfield, IN, USA) and antibiotic (7 g/ton on DM basis; tylosin, Tylan, Elanco) from day 0 to 180. Moreover, GPT+ steers received an in-feed beta-adrenergic agonist during the last 42 d of the feeding period (27 g/ton on DM basis; ractopamine hydrochloride, Actogain, Zoetis), allowing for a 2 d withdrawal period prior to harvest. The GPT− steers did not receive any of the above-listed GPT.

Regardless of management treatment and cattle sub-species, all steers were vaccinated with 2 mL Bovi-Shield GOLD 5 (Bovine rhinotracheitis-virus diarrhea-parainfluenza 3-respiratory syncytial virus vaccine, Zoetis), 2 mL Ultrachoice 8 (clostridial vaccine, Zoetis), Dectomax (doramectin, Zoetis), and orally drenched with Valbazen (albendazole, Zoetis) within 48 h post arrival to the feedlot. All cattle received a radio frequency identification tag (Allflex, USA Inc., Dallas, TX, USA) placed in the animal’s left ear on the same processing date.

Steers were fed for 180 d in two consecutive phases. In Phase 1 (day 0 to 83), steers were housed in 10-hd research pens (7 m × 40 m). Steers were blocked by weight and randomly assigned to a pen, where the pen was considered the experimental unit (10 steers/pen; 5 pens/treatment). Each pen was equipped with a concrete feed bunk (30 cm/steer), a 3 m × 7 m concrete bunk pad, and an automatic waterer (model no. C250, Cancrete Cattle Waterer, Advanced Agri-Direct Inc., York, NE, USA). In Phase 2 (day 84 to 180), steers receiving the same treatment were moved and randomly assigned to 1 of the 4 Climate Smart Research Pens (15 m × 43 m) for the remaining 96 d of the experimental period, where the experimental unit was considered the individual animal (50 steers/treatment). Each Climate Smart Research Pen housed 50 steers/pen (1 pen/treatment), equipped with one GreenFeed automated head chamber system (AHCS, C-Lock, Rapid City, SD, USA), five SmartFeed bunk systems (C-Lock, Rapid City, SD, USA), two automatic waterers, a concrete bunk pad (15 m × 3 m), and a metal roof (15 m × 3 m) covering the SmartFeed bunk systems, AHCS, and approximately 7% of the pen surface.

Steers were monitored daily throughout the experimental period by trained personnel who evaluated fresh feed and water allocation, cattle health, locomotion, and clinical signs of disease. Steers exhibiting signs of respiratory disease, confirmed by a licensed on-site veterinarian, were removed from the pen to assess rectal body temperature and body weight (BW). Cattle with body temperatures greater than 39.4°C were classified as clinically ill. All steers deemed clinically ill by the licensed on-site veterinarian were treated according to the appropriate treatment protocol prescribed by the on-site licensed veterinarian and immediately returned to their pen of origin. Any cattle deemed clinically ill or treated by the licensed on-site veterinarian were removed from the experiment. All treatments were managed according to the same animal health protocols. Following the completion of the experimental period, a total of eight steers were removed from data analysis due to injury or illness: four BT GPT+ and four BT GPT−.

### Feed management and intake

All steers were transitioned to the final finishing diet after a 21 d adaptation from a starter diet to a finishing diet, including two intermediate step-up diets (Table 2). Since some GPT were delivered in the diet, specific feed mixing and delivery protocols were implemented to prevent cross-contamination between the two rations throughout the experimental period. At the start of each feeding, the feed truck mixer was purged by mixing and delivering a standard ration to clean the equipment. Following this cleaning procedure, the GPT− ration was batched, mixed, and delivered first, after which the GPT+ ration was batched, mixed, and delivered.

**Table 2.** Dietary ingredients and analyzed chemical composition of the diets

Item^1^	Diet			
	Starter	Step 1	Step 2	Finish
***Ingredient, % DM***				
**Steam-flaked corn**	30.0	44.0	55.0	65.0
**Corn silage**	50.0	40.0	30.0	20.0
**Distillers grains w/solubles**	13.5	9.5	8.5	7.0
**Molasses-based supplement**	3.3	3.3	3.3	4.0
**Mineral and vitamin supplement**	3.2	3.2	3.2	4.0
***Chemical Composition, DM basis* ^2^**				
**DM, %**	54.6	53.1	60.3	64.3
**CP, %**	15.7	16.2	14.2	14.4
**ADF, %**	11.2	9.4	8.1	7.8
**NDF, %**	23.3	21.2	18.6	17.2
**Lignin, %**	1.8	1.6	1.9	1.3
**Starch, %**	43.1	46.3	52.8	53.2
**Ash, %**	7.0	5.6	3.9	4.5
**TDN, %**	78.0	80.5	82.0	82.5
**NE_m_, Mcal/kg**	1.91	1.98	2.04	2.06
**NE_g_, Mcal/kg**	1.27	1.33	1.38	1.40
**GE, Mcal/kg**	4.42	4.49	4.45	4.44

1 DM, dry matter; CP, crude protein; ADF, acid detergent fiber; NDF, neutral detergent fiber; TDN, total digestible nutrients; NE_m_, net energy for maintenance; NE_g_, net energy for gain; GE, gross energy.

2 Dietary composition was determined via wet chemistry.

Feed samples of each treatment ration were collected weekly, dried at 65 °C for a minimum of 48 h to determine DM and ground to pass through a 2 mm screen (Wiley Mill, Model 4; Arthur H. Thomas Co., Philadelphia, PA, USA) and preserved at room temperature. After the completion of the experimental period, feed samples of each treatment ration were composited and analyzed at a commercial laboratory using a wet chemistry package (Ration Balancer Plus with Gross Energy and Starch, Dairy One, Ithaca, NY, USA). Acid detergent fiber analysis was conducted following ANKOM Technology Method 14, and neutral detergent fiber analysis was conducted following ANKOM Technology Method 15 (ANKOM Technology, Macedon, NY, USA). Analysis of lignin was determined following ANKOM Technology Method 9 using a DaisyII Incubator (ANKOM Technology, Macedon, NY, USA). Crude protein analysis was determined via combustion and determination of ash used the AOAC Official Method 942.05- Ash for Animal Feed. Gross energy was determined using bomb calorimetry. Due to no significant difference in chemical analysis of the two treatment rations, average composition is reported.

During Phase 1, slick-bunk management was utilized to manage feed delivery to the feed bunk, and feed intake data was averaged daily on an animal-per-pen basis. Daily feed allocation per pen was divided by steer/pen, averaged by week, and multiplied by the percent DM of the total mixed ration (TMR) to determine weekly dry matter intake (DMI). Cattle removed from the study were documented to ensure DMI was calculated on a steer/pen basis and adjusted for the number of animals remaining in each pen following the removal of a steer from the study. During Phase 2, individual animal feed intake was determined utilizing SmartFeed bunk systems, and ad libitum bunk management was utilized to manage feed delivery. All cattle underwent a 7 d acclimation period to the SmartFeed bunk systems before the initiation of data collection. Feed intake data collected during the acclimation period were not used in the data analysis. The SmartFeed bunk systems were calibrated at the beginning and end of the experimental period and every 7 d during the experimental period. Following the conclusion of the experimental period, raw feeding events were downloaded from the manufacturer’s online software. Feeding events that were flagged by the manufacturer (e.g., unallocated feed or nonsensical eating rate), exceeded 3,600 s in duration, and/or were greater than 10 kg mass difference were omitted from data analysis (approximately 47,000 events removed in total). Feeding events were then summed by day and averaged by week to determine weekly as-fed feed intake. As-fed feed intake was converted to DMI by multiplying by the percent DM of the TMR. To determine the average DMI for each phase, weekly DMI was averaged over the feeding period. During Phase 2 of the experimental period, DMI from alfalfa pellet drops from the AHCS were added to the TMR daily intake to determine total daily DMI. In addition to total daily DMI in Phases 1 and 2, DMI as a percentage of BW (DMI_BW_) was calculated for each phase using Equation 1.


(1) $${\mathrm{DMI}}_{BW},\;\%\;BW=\frac{\mathrm{DMI},\;kg}{BW,\;kg}\;\times 100$$

### Growth performance

Steers were individually weighed using a hydraulic squeeze chute (Model no. SHEW10497, Silencer, Moly Manufacturing Inc., Lorraine, KS, USA) equipped with an electronic weighing system (ID5000, Tru-Test Inc., Mineral Wells, TX, USA) on two consecutive days at the beginning of the experiment (day − 1 and 0) to determine IBW and at the end (day 179 and 180) of the experimental period to determine final BW (FBW). In addition to IBW and FBW, steers were weighed every 28 d during the experimental period. Experimental mid-point BW, collected on day 84, was used as the FBW for Phase 1 and IBW for Phase 2. Weight data collected were reduced by 4% to account for digestive tract fill (i.e., shrunk body weights [SBW]; NASEM, 2016). The SBW from each time point, every 28 d, was used to obtain cattle average daily gain (ADG) by subtracting the SBW of the previous time point from the SBW of the current time point and then dividing by the number of days between the two weight collection time points.

### Gas flux

Each climate smart research pen contained one AHCS for the collection of gas flux (i.e., CH_4_, CO_2,_ and H_2_ emissions, and O_2_ consumption). Steers were exposed to the AHCS during an acclimation period of 7 d before the initiation of data collection. The headbox extension panels (i.e., wings) were placed on the AHCS system on day 3 of the acclimation period. On day 5 of the acclimation period, cattle panels were used to create an alley in front of the AHCS to ensure only one animal at a time had access to the AHCS. Measurements collected during the acclimation period were not included in the data analysis. Throughout the study, steers were allowed to visit the AHCS system up to 6 visits/d, with a minimum of 4 h between each visit. The AHCS was programmed to allow consumption of up to six drops of alfalfa pellets per visit (approximately 38 g as fed/drop), with drops occurring every 30 s while a steer was utilizing the AHCS system. These configuration settings encouraged an even distribution of AHCS visits for each steer throughout the day, capturing the diurnal variation in gas flux, and ensuring each steer remained at the AHCS for a minimum duration of 2 min during gas flux sample collection (Vargas et al., 2024).

To ensure proper functioning and performance of the AHCS, CO_2_ recovery tests were performed at the beginning, end, and every 28 d throughout the experimental period to gravimetrically calibrate the AHCS. Additionally, zero and span calibrations of the CH_4_, CO_2_, H_2_, and O_2_ gas analyzers were performed every 3 d via an autocalibration system located onboard the AHCS. Raw collection data was validated by the AHCS manufacturer, which included checking head proximity, visit length, and airflow, as well as providing corrections for wind. Data was excluded when the length of the visit was less than 2 min, greater than 8 min, and the airflow was less than 26 L/s (Arthur et al., 2017; Gunter and Beck, 2018). Steers were excluded from the analysis of data if the total number of visits collected throughout Phase 2 was less than 40 (Beck et al., 2024). Of the 192 steers, excluding the 8 steers that were removed from the trial for health-related reasons, 149 steers met the filtering requirements to be included in the gas flux data analysis (44 BT GPT+, 37 BI GPT+, 40 BT GPT−, and 28 BI GPT−). For the steers that met the filtering requirements, the average number of gas flux visits per treatment was as follows: 151 visits/steer for BT GPT+, 148 visits/steer for BI GPT+, 109 visits/steer for BT GPT−, and 71 visits/steer for BI GPT−.

In addition to the collection of CH_4_, CO_2_, H_2_, and O_2_ gas flux, CH_4_ conversion rate (*Y*_m_) was calculate as the energy emitted as CH_4_ relative to gross energy intake (GEI), CH_4_ yield (MY) was calculated as the amount of CH_4_ relative to DMI, and CH_4_ intensity (EI) was calculated as the amount of CH_4_ relative the ADG in Phase 2, using Equation 2, 3, and 4, respectively.


(2) $$Ym,\;\%\;\mathrm{GEI}=\;\frac{\mathrm{Energy}\;\mathrm{Emitted}\;as\;{\mathrm{CH}}_{4},\;\mathrm{Mcal}/\mathrm{kg}}{\mathrm{GEI},\;\mathrm{Mcal}/\mathrm{kg}}\times 100$$

(3) $$MY,\frac{g}{\mathrm{kg}}=\frac{{\mathrm{CH}}_{4},\;g}{\mathrm{DMI},\;\mathrm{kg}}$$

(4) $$EI,\frac{g}{\mathrm{kg}}=\frac{{\mathrm{CH}}_{4},\;g}{\mathrm{ADG},\;\mathrm{kg}}$$

### Carcass characteristics

Following the collection of weights on day 180, steers were shipped to a commercial abattoir in Fort Morgan, Colorado, approximately 150 km from the Colorado State University Agricultural Research, Education, and Development Center, for harvest. Harvest data were collected by a third party (Diamond T Livestock Services, Inc., Alliance, NE, USA). On the same day as slaughter, HCW, and liver abscess prevalence (scored as 0, A−, A, and A+; Brown et al., 1975) were assessed. Carcasses were chilled for 24 h at −4°C. Approximately 24 h after slaughter, the carcasses were ribbed between the 12th and 13th ribs, and carcass data were assessed via cameras, including 12th rib backfat thickness (FT), longissimus muscle area (LMA), and marbling score. Yield grade (YG) was calculated using the United States Department of Agriculture (USDA) YG equation with a standard kidney, pelvic, and heart fat percentage of 2.0 (USDA, 1997; Boykin et al., 2017). The USDA YG and quality grade (QG) for each carcass was assigned by a USDA grader at the commercial abattoir. The “No Roll” QG indicates carcasses that did not receive an official USDA QG or YG at the commercial abattoir due to quality below the USDA Select grade; however, these carcasses were still inspected for wholesomeness and safety for human consumption. All carcass characteristics were assessed for “No Roll” carcasses and included in data analysis. Dressing percentage (DP) was calculated using Equation 5.


(5) $$\mathrm{Dressing}\;\mathrm{percentage},\;\%=\frac{\mathrm{HCW},\;kg}{\mathrm{Final}\;\mathrm{SBW},\;kg}\times 100$$

### Statistical analysis

In Phase 1, the experimental design was a randomized complete block design with a 2 × 2 factorial arrangement of the treatments. Data were analyzed with R (R Core Team, 2021, v. 4.4.1) software using the lmer() function within the lme4 package to construct a linear mixed-effects model. The model diagnostics included testing for normal distribution of the error residuals via visual assessment of the histogram and Q-Q plot for each variable, as well as formally conducting the Shapiro-Wilk test, homogeneity of variance via visual assessment of the residual versus fitted values plot, and independence among observations which was ensured during the study design. The assumptions were adequately held.

For Phase 1, the fitted model was:


$$Y_{\mathrm{ijkl}}=µ+S_{i}+T_{j}+{\left(ST\right)}_{ij}+W_{k}+B_{l}+E_{\mathrm{ijkl}}$$

where *Y_ijkl_* is the response variable; *S_i_* is the fixed effect of sub-species; *T_j_* is the fixed effect of treatment; (*ST*)_*ij*_ is the interaction between sub-species and treatment; *W_k_* is the fixed effect of IBW of the phase; *B_l_* is the random effect of block; and *E_ijkl_* is the experimental error.

The experimental design of Phase 2 was a completely randomized design with a 2 × 2 factorial arrangement of treatments. Data were analyzed with R (R Core Team, 2021, v. 4.4.1) software using the aov() function within the base R package to construct a linear effects model. The model diagnostics included testing for normal distribution of the error residuals, homogeneity of variance, and independence among observations using methodologies previously described. The assumptions were adequately held.

For Phase 2, the fitted model was:


$$Y_{\mathrm{ijk}}=µ+S_{i}+T_{j}+{\left(ST\right)}_{ij}+W_{k}+E_{\mathrm{ijk}}$$

where *Y_ijk_* is the response variable; *S_i_* is the fixed effect of sub-species; *T_j_* is the fixed effect of treatment; (*ST*)_*ij*_ is the interaction between sub-species and treatment; *W_k_* is the fixed effect of IBW of the phase; and *E_ijk_* is the experimental error.

Categorical data distributions were analyzed by logistic regression. The model was constructed using the glm() function of the base R package. Least-squares means were derived using the emmeans package in R, with Tukey’s HSD adjustment. For Phase 1, pen was the experimental unit, and for Phase 2, individual animal was the experimental unit. Differences were declared significant at *P *≤ 0.05.

## Results

### Growth performance

In Phase 1, no interaction (*P *> 0.23) was observed between cattle sub-species × treatment for growth performance variables (Table 3). However, IBW, differed (*P *< 0.01) by cattle sub-species, with BI steers being heavier than BT steers at the beginning of the experiment. Moreover, BT cattle had greater (*P *< 0.01) DMI, DMI_BW_, G:F, ADG, and FBW compared to BI steers. Although IBW was different between cattle sub-species, it did not differ (*P *= 0.98) by management treatment. No treatment effect (*P *= 0.19) was observed for DMI. However, GPT+ steers had greater (*P *≤ 0.02) FBW, ADG, and G:F compared to GPT− steers for Phase 1.

**Table 3.** Growth performance of finishing *Bos taurus taurus* and *Bos taurus indicus* steers managed with and without the use of growth-promoting technologies during Phase 1 (0 to 83 d)

Item^1^	*Bos taurus indicus*		*Bos taurus taurus*		SEM^3^	S^4^	T^4^	S × T^4^
	GPT−^2^	GPT+^2^	GPT−^2^	GPT+^2^				
***n*, pen**	5	5	5	5	–	–	–	–
**IBW, kg**	364	364	323	322	8.5	<0.01	0.98	1.00
**FBW, kg**	417	429	502	521	3.9	<0.01	0.02	0.23
**ADG, kg**	0.88	1.02	1.88	2.11	0.046	<0.01	0.01	0.24
**DMI, kg/d**	7.1	6.8	9.9	9.6	0.14	<0.01	0.19	0.64
**DMI_BW_, % of BW**	1.86	1.78	2.38	2.24	0.037	<0.01	0.09	0.35
**G:F**	0.125	0.149	0.191	0.223	0.0071	<0.01	<0.01	0.38

1 IBW, initial body weight; FBW, final body weight; ADG, average daily gain; DMI, dry matter intake; DMI_BW_, dry matter intake according to the body weight; G:F, gain to feed ratio.

2 Experimental management treatment; GPT−, managed without the use of growth-promoting technologies; GPT+, cattle managed with the use of growth-promoting technologies.

3 SEM, standard error of the mean.

4 S, main effect cattle sub-species; T, main effect management treatment; S × T, interaction between cattle sub-species and management treatment.

In Phase 2, no interaction (*P *> 0.14) was observed between cattle sub-species × treatment for IBW, ADG, and FBW (Table 4). However, an interaction (*P *≤ 0.04) was observed for DMI, DMI_BW_ and G:F. The use of GPT resulted in a greater increase in DMI between GPT− and GPT+ for BI steers compared to BT steers. Similarly, DMI_BW_ had a cattle sub-species × treatment interaction (*P *< 0.01) where a greater magnitude of difference was observed in DMI_BW_ between GPT− and GPT+ for BI steers compared to BT steers. Moreover, G:F had a cattle sub-species × treatment interaction (*P *< 0.01) where a greater increase in feed efficiency was observed in BI steers between GPT+ and GPT− than BT counterparts. Furthermore, Phase 2 IBW, ADG, and FBW differed (*P *< 0.001) by cattle sub-species, with BT steers exhibiting greater IBW, ADG, and subsequent FBW than BI steers. Moreover, IBW, ADG, and FBW differed (*P *< 0.01) by treatment with GPT+ steers starting at heavier body weights, gaining more, and finishing at heavier FBW than GPT− steers.

**Table 4.** Growth performance of finishing *Bos taurus taurus* and *Bos taurus indicus* steers managed with and without growth-promoting technologies during Phase 2 (84 to 180 d)

Item^1^	*Bos taurus indicus*		*Bos taurus taurus*		SEM^3^	S^4^	T^4^	S × T^4^
	GPT−^2^	GPT+^2^	GPT−^2^	GPT+^2^				
***n*, animal**	50	50	46	46	–	–	–	–
**IBW, kg**	441	453	477	497	4.8	<0.01	<0.01	0.37
**FBW, kg**	538	583	603	637	4.2	<0.01	<0.01	0.14
**ADG, kg**	0.74	1.22	1.42	1.78	0.044	<0.01	<0.01	0.14
**DMI, kg/d**	7.2^c^	8.0^b^	11.2^a^	11.3^a^	0.18	<0.01	<0.01	0.04
**DMI_BW_, % BW**	1.43^b^	1.53^b^	2.08^a^	2.03 ^a^	0.029	<0.01	0.43	<0.01
**G:F**	0.101^c^	0.152^a^	0.127^b^	0.157^a^	0.0042	<0.01	<0.01	<0.01

1 IBW, initial body weight; FBW, final body weight; ADG, average daily gain; DMI, dry matter intake; DMI_BW_, dry matter intake according to the body weight; G:F, gain to feed ratio.

2 Experimental management treatment; GPT−, managed without the use of growth-promoting technologies; GPT+, cattle managed with the use of growth-promoting technologies.

3 SEM, standard error of the mean.

4 S, main effect cattle sub-species; T, main effect management treatment; S × T, interaction between cattle sub-species and management treatment.

a, b, c Means in the same row within main effect that do not have a common superscript letter differ, *P *≤ 0.05.

### Carcass characteristics

No interaction (*P *≥ 0.07) was observed between cattle sub-species × treatment for DP, FT, LMA, calculated YG, or liver abscess prevalence (Table 5). However, an interaction (*P *≤ 0.03) was observed for HCW and marbling score. For HCW a greater magnitude of difference was observed between GPT− and GPT+ for BI steers compared to BT. Moreover, the use of GPT in BT steers reduced marbling scores compared to GPT−, while no difference was observed in marbling score for BI cattle between the two treatments. Cattle sub-species differed (*P *< 0.01) for DP, FT, LMA, and calculated YG; however, no difference (*P *≥ 0.21) between cattle sub-species was observed for liver abscess prevalence. Steers of BI origin exhibited greater DP and LMA, while BT steers had greater FT and calculated YG. Management treatment did not differ (*P *≥ 0.30) for DP, FT, calculated YG, or liver abscess prevalence, but differed (*P *< 0.01) for LMA. The use of GPT yielded greater LMA than when managed without GPT.

**Table 5.** Carcass data from *Bos taurus taurus* and *Bos taurus indicus* steers managed with and without the use of growth-promoting technologies following a 180-d feeding period

Item^1^	*Bos taurus indicus*		*Bos taurus taurus*		SEM^3^	S^4^	T^4^	S × T^4^
	GPT−^2^	GPT+^2^	GPT−^2^	GPT+^2^				
***n*, animal**	49	49	46	46	–	–	–	–
**HCW, kg**	345^c^	376^b^	373^b^	390^a^	3.2	<0.01	<0.01	0.02
**DP, %**	64.1	64.4	61.8	61.2	0.28	<0.01	0.89	0.07
**FT, cm**	1.22	1.11	1.56	1.38	0.061	<0.01	0.14	0.57
**LMA, cm^2^**	85.2	92.0	79.7	84.9	1.33	<0.01	<0.01	0.42
**Calculated YG**	2.78	2.59	3.62	3.34	0.096	<0.01	0.11	0.63
**Marbling Score^5^**	382^c^	380^c^	552^a^	500^b^	15.1	<0.01	0.09	0.03
**Liver Abscess Prevalence,^6^ %**								
**0**	85.7	71.4	67.4	73.9	6.91	0.21	0.50	0.08
**A−**	4.1	6.1	6.5	8.7	4.15	0.48	0.55	0.92
**A**	0.0	4.1	2.2	2.2	2.83	0.95	0.30	0.19
**A+**	10.2	18.4	23.9	15.2	6.29	0.33	1.00	0.12
**Total Abscess Prevalence**	14.3	28.6	32.6	26.1	6.91	0.21	0.50	0.08

1 HCW, hot carcass weight; DP, dressing percentage; FT, Back fat measured between the 12th and 13th rib after 24 h chill; LMA, longissimus muscle area; YG, yield grade.

2 Experimental management treatment; GPT−, managed without the use of growth-promoting technology, GPT+, cattle managed with the use of growth-promoting technology.

3 SEM, standard error of the mean.

4 S, main effect cattle sub-species; T, main effect management treatment; S × T, interaction between cattle sub-species and management treatment.

5 Marbling score scale: 300 = slight, 400 = small, 500 = modest.

6 0,  non-abscessed liver; A-minus (A−), one or two small abscesses; A, two to four small, active abscesses; A-plus (A+), one or more large, active abscesses, including any open and adhered abscesses.

a, b, c Means in the same row within main effect that do not have a common superscript letter differ, *P *≤ 0.05.

When assessing YG and QG percentage distributions, no interactions (*P *≥ 0.20) were observed between cattle sub-species × treatment (Table 6). However, the percentage distribution of YG differed (*P *≤ 0.02) by cattle sub-species for YG 1, 2, 3, 4, and No Roll carcasses. Carcasses of BI steers had a greater proportion of No Roll, YG 1, and YG 2 when compared to BT. However, BT had a greater proportion of YG 3 and YG 4 carcasses. Additionally, the percentage distribution of USDA QG differed (*P *< 0.01) by cattle sub-species for Prime, Choice, Select, and No Roll grades. Carcasses of BI steers had a greater (*P *< 0.01) proportion of No Roll and Select QG, while Choice and Prime QG were observed in greater (*P *< 0.01) proportions for BT. Moreover, the percentage distribution for USDA YG differed (*P *≤ 0.04) by treatment for YG 3 and 4 but did not differ (*P ≥ *0.30) by treatment for YG 1, 2, or No Roll, or USDA QG. A greater proportion of YG 3 carcasses were observed for GPT+ compared to GPT−, and a greater proportion of YG 4 carcasses were observed in GPT− compared to GPT+.

**Table 6.** United States Department of Agriculture yield and quality grade distribution for *Bos taurus taurus* and *Bos taurus indicus* steers managed with and without the use of growth-promoting technologies following a 180-d feeding period

Item^1^	*Bos taurus indicus*		*Bos taurus taurus*		SEM^3^	S^4^	T^4^	S × T^4^
	GPT−^2^	GPT+^2^	GPT−^2^	GPT+^2^				
***n*, animal**	49	49	46	46	–	–	–	–
**USDA YG, %**								
** YG 1**	16.3	16.3	0.0	0.0	5.28	<0.01	1.00	1.00
** YG 2**	57.1	44.9	4.4	8.7	7.02	<0.01	0.47	0.20
** YG 3**	20.4	32.7	52.2	67.4	7.37	<0.01	0.04	0.99
** YG 4**	4.1	0.0	43.5	23.9	7.31	<0.01	0.02	0.25
** No Roll**	2.0	6.1	0.0	0.0	3.42	0.02	0.30	1.00
**USDA QG, %**								
** Prime**	0.0	0.0	6.5	4.4	3.64	<0.01	0.64	1.00
** Choice**	28.6	28.6	93.5	95.7	6.45	<0.01	0.84	0.68
** Select**	59.2	63.3	0.0	0.0	7.02	<0.01	0.68	1.00
** No Roll**	12.2	8.2	0.0	0.0	4.68	<0.01	0.50	1.00

1 USDA Yield Grade (YG); USDA Quality Grade (QG), Quality grade marketing category determined by expert marbling score: USDA Prime, Slightly Abundant^0^ to Abundant^99^; USDA Choice, Small^0^ to Moderate^99^; USDA Select, Slight^0^ to Slight^99^; No Roll, Carcass was not designated an official USDA QG due to intramuscular fat classification lower than USDA Select.

2 Experimental management treatment; GPT−, managed without the use of growth-promoting technology; GPT+, cattle managed with the use of growth-promoting technology.

3 SEM, standard error of the mean.

4 S, main effect cattle sub-species; T, main effect management treatment; S × T, interaction between cattle sub-species and management treatment.

### Gas flux

No interaction (*P *≥ 0.08) was observed between cattle sub-species × treatment for CH_4_, CO_2_, H_2_, Y_m_, or MY (Table 7). However, an interaction (*P *≤ 0.01) was observed between cattle sub-species × treatment for O_2_ and EI. Consumption of O_2_ was greater for GPT+ compared to GPT− for BT steers, while no difference was observed between treatments for BI steers. Additionally, when GPT was utilized for BI steers EI decreased compared to BI steers managed without GPT; however, no effect was observed between treatments for BT steers. Total daily emissions of CH_4_ differed (*P *< 0.01) by cattle sub-species and were greater for BT steers than BI steers. Additionally, emissions of CO_2_ and H_2_ differed (*P *< 0.01) by cattle sub-species, with BT steers exhibiting greater emissions of CO_2_ and H_2_ than BI steers. Moreover, *Y_m_* and MY, were greater (*P *< 0.01) for BI steers than for BT steers, meaning more CH_4_ was emitted per unit of GEI and DMI by BI steers than BT steers, respectively. Gas flux measurements did not differ (*P *≥ 0.09) by treatment.

**Table 7.** Gas flux of finishing *Bos taurus taurus* and *Bos taurus indicus* steers managed with and without the use of growth-promoting technologies during Phase 2 (84 to 180 d)

Item^1^	*Bos taurus indicus*		*Bos taurus taurus*		SEM^3^	S^4^	T^4^	S × T^4^
	GPT−^2^	GPT+^2^	GPT−^2^	GPT+^2^				
***n*, animal**	28	37	40	44	–	–	–	–
**AHCS visits, n**	71	148	109	151	–	–	–	–
**DMI, kg/d**	7.3^c^	8.1^b^	11.1^a^	11.4^a^	0.22	<0.01	0.03	0.04
**ADG, kg**	0.73	1.20	1.43	1.81	0.057	<0.01	<0.01	0.31
**CH_4_, g/d**	145	153	162	165	5.8	<0.01	0.09	0.67
**CO_2_, g/d**	8000	7788	10051	10174	165.0	<0.01	0.42	0.21
**O_2_, g/d**	5504^c^	5598^c^	6743^b^	7077^a^	109.0	<0.01	<0.01	0.01
**H_2_, g/d**	1.06	1.09	1.26	1.09	0.076	<0.01	0.26	0.09
**Y_m_, % GEI**	6.07	5.65	4.30	4.43	0.193	<0.01	0.63	0.08
**MY, g CH_4_/kg DMI**	20.2	18.8	14.4	14.8	0.63	<0.01	0.61	0.08
**EI, g CH_4_/kg ADG**	244.8^a^	124.5^b^	121.0^b^	98.9^b^	17.5	<0.01	<0.01	<0.01

1 AHCS, automated head chamber system; DMI, dry matter intake; ADG, average daily gain; CH_4_, methane; CO_2_, carbon dioxide; O_2_, oxygen; H_2_, hydrogen; Y_m_, CH_4_ emitted as a percent of gross energy intake (GEI); MY, methane yield; EI, emissions intensity.

2 Experimental management treatment; GPT−, managed without the use of growth-promoting technology; GPT+, cattle managed with the use of growth-promoting technology.

3 SEM, standard error of the mean.

4 S, main effect cattle sub-species; T, main effect management treatment; S × T, interaction between cattle sub-species and management treatment.

a, b, c Means in the same row within main effect that do not have a common superscript letter differ, *P *≤ 0.05.

## Discussion

### Growth performance

Instances of extreme heat have become more frequent and severe across most land regions since the 1950s, while cold extremes have become less frequent and severe (IPCC, 2021). However, the migration of the US cattle population to more temperate regions, which may experience both hot and cold extremes, garners research assessing the sustainability of cattle selection and management adaptations to ensure the resiliency of the supply chain (Fancher et al., 2025). While BI breeds have received attention regarding their utilization in environments that are experiencing increased ambient temperatures, these breeds have historically received criticism from the beef feedlot and packing industries, primarily regarding poor growth performance and carcass characteristics (Huffman et al., 1990). Another consideration regarding BI breeds is their efficiency of utilizing poor quality diets as opposed to good quality diets (Butler et al., 1956b; Arthur et al., 1994; Chaokaur, 2015), which is a production challenge to consider in the application of increasing the proportion of BI cattle in feedlot systems where good quality diets (i.e., grain-based rations) are commonly fed.

When considering BI and BT comparative research trials, few have conducted comparative evaluations of straight-bred BT and BI cattle, likely because it has been recommended that the greatest advantage of BI cattle lies in the crossbreeding programs in the south and southwest regions of the US where the crosses are well adapted to the hot, humid climate (Paschal et al., 1995). Preceding literature would indicate that BT cattle consume greater quantities of feed (Boyles and Riley, 1991; Ferrell and Jenkins, 1998) with greater feed efficiency (Boyles and Riley, 1991) than BI counterparts, generally corresponding to greater growth rates in BT cattle (Butler et al., 1956b; Lunt et al., 1985; Boyles and Riley, 1991). Boyles and Riley (1991) reported that following a 184 d feeding period in cold environmental conditions, Angus steers gained 17 kg more than Brahman-Angus steers, resulting in Angus steers having greater ADG throughout the feeding period; however, ADG of Angus steers diminished slightly over time compared to Brahman-Angus steers. Moreover, Boyles and Riley (1991) reported that BI × BT steers consumed 0.2% less feed as a percentage of BW than Angus steers. Similarly, Butler et al. (1956b) reported that BT steers had greater ADG than BI × BT steers, and Lunt et al.(1985) observed that Angus steers finished at heavier BW than Brahman steers following a 168 d feeding period on a commercial finishing diet. These observations align with the present study. In the present study, BI steers entered Phase 1 with an IBW approximately 12% greater than BT steers. Nonetheless, by the end of the phase, BT steers weighed approximately 19% more, reflecting their greater ADG, DMI, and G:F. Given that BI steers were approximately 42 kg heavier at study initiation, it is possible they were older than BT steers, despite all cattle being sourced as yearlings. Importantly, IBW was included in the statistical model to account for such differences. Furthermore, growth performance observations in Phase 2 were consistent with Phase 1, although responses to GPT diverged between sub-species for DMI and G:F.

To the authors’ knowledge, previous comparative studies of BT and BI steers have not specifically reported differences in illness or removal rate. In the present study, 8% of BT steers (4% GPT+ and 4% GPT−) were removed due to illness, whereas no BI steers were removed. From a systems perspective, this observation has potential implications for overall production efficiency and warrants further investigation. Notably, despite the 8% removal rate, BT steers still yielded more total live weight than BI steers (28,520 vs. 28,025 kg), indicating that growth performance advantages of BT cattle remained evident. Nevertheless, the higher removal rate in BT steers may offset some of these advantages under commercial conditions, particularly in environments where health and adaptability challenges are more pronounced. Moreover, many factors influence the health of a feedlot steer such as preconditioning (Duff and Galyean, 2007). Since steers in this study were sourced from different regions of the US, differences in preconditioning and prior management could potentially contributed to the observed removal rates. Moreover, it should be acknowledged that the present study was conducted at 1,557.5 m above sea level. It has been reported that in Angus cattle even moderate elevations (1,200 to 1,600 m) can cause physiological alterations that predispose cattle to disease (Pauling et al., 2018). Future investigations should further examine breed-specific differences in altitude-induced pathophysiological responses that may influence feedlot health and survival.

Recent research has suggested that different cattle breed types may respond differently to GPT (Reichhardt et al., 2021; Rivero et al., 2021). However, determining optimal cattle management with GPT across different cattle sub-species in feedlots in temperate climates is needed (Reichhardt et al., 2023). When investigating cattle of BI × BT and BT origin in temperate environmental conditions, Reichhardt et al. (2023) reported steers receiving an anabolic implant had greater DMI and G:F, corresponding to greater ADG. It is well accepted that of the GPT available in cattle production systems, anabolic implants increase DMI, G:F, and ADG of feedlot steers (Duckett and Pratt, 2014; Smith and Johnson, 2020). Duckett and Pratt (2014) observed that cattle managed with anabolic implants consumed 6% more feed with 6% greater feed efficiency, resulting in an 18% improvement in ADG. Similarly, Fox et al. (1992) reported that cattle not receiving an anabolic implant can be expected to have DMI 6% less than implanted cattle. When considering ionophores, Duffield et al. (2012) conducted a meta-analysis investigating the effect of monensin on beef cattle growth performance and observed a 6.4% increase in G:F, a 3% decrease in DMI, and a 2.5% increase in ADG. Furthermore, when considering beta-adrenergic agonists, ractopamine hydrochloride supplementation has consistently been demonstrated to increase BW, ADG, and G:F when compared to non-supplemented beef steers, regardless of use in combination with anabolic implants, tylosin phosphate, and/or monensin (Bryant et al., 2010, 2020; Scramlin et al., 2010; Genther-Schroeder et al., 2016). The present study observed a 33% improvement in ADG and 31% improvement in G:F between GPT+ and GPT− steers, which is similar to the 37.8% improvement in ADG and 33.3% improvement in G:F between BT steers receiving tylosin phosphate, monensin, an anabolic implant, and a beta-adrenergic agonist versus not receiving any GPT (Maxwell et al., 2015). A difference in the magnitude of response when multiple GPT are utilized may suggest the potential for an additive or synergetic response when cattle are managed with a combination of GPT. Additive responses are plausible considering different mechanisms of actions between various GPT, since some GPT (i.e., ionophores and antibiotics) alter the rumen microbial composition, while others (i.e., anabolic implants and beta-adrenergic agonists) modulate post-absorptive metabolism (NASEM, 2016). Despite the use of multiple GPT in the present study and the inability to identify how each GPT may be influencing the observed growth performance, observations clearly demonstrate that the use of GPT resulted in greater FBW, ADG, and G:F in feedlot steers. However, the present study also highlights that cattle of differing sub-species do not elicit the same growth performance response when using GPT, suggesting that GPT use may be more valuable to use in certain sub-species of cattle, which may require further investigation.

On a total daily basis, BT steers consumed more feed than BI steers during both Phase 1 (9.8 vs. 7.0 kg/d) and Phase 2 (11.3 vs. 7.6 kg/d). Within BT steers, average DMI did not differ between those managed without or with growth-promoting technologies (GPT− and GPT+; 11.3 kg/d). In a model-based analysis, Crawford et al. (2022) reported that feedlot cattle finished without GPT had a mean DMI of 8.9 kg/d at a FBW of 602 kg, compared with 9.0 kg/d at an FBW of 655 kg for cattle managed with GPT (i.e., anabolic implants, ionophores, and β-adrenergic agonists). Similarly, Stackhouse-Lawson et al. (2013) observed no significant differences in DMI between steers of comparable FBW managed without GPT (10.2 kg/d) and with GPT (10.6 kg/d; i.e., anabolic implants, ionophores, and β-adrenergic agonists). Compared to Crawford et al. (2022) and Stackhouse-Lawson et al. (2013), the greater DMI observed in the present experiment may reflect increased maintenance energy requirements associated with winter feeding conditions (NASEM, 2016).

When considering differences in growth performance and efficiency between BI and BT steers in winter conditions, the greater surface area of BI cattle could be detrimental to conserving heat (Boyles, 1985). For most animals reared in the US in outside environments, maintenance energy requirements will decrease when the environmental temperature is greater than 20°C but increase when less than 20°C (NASEM, 2016; BR-CORTE, 2023). Despite recommendations that BI cattle require approximately 10% less energy than BT beef cattle (NASEM, 2016), Boyles (1985) concluded that net energy for maintenance (NE_m_) requirements were reduced by approximately 6% for BI × BT steers compared to BT steers in thermoneutral conditions; but increased 9% for BI × BT steers compared to BT steers in cold stress conditions. Therefore, when more energy is partitioned to maintaining homeothermy, less energy is available for gain. Moreover, when an energy-dense diet is fed, BT steers generally gain weight more efficiently and at a faster rate than BI steers, due to greater feed consumption relative to energy requirements for BT steers compared to BI steers (NASEM, 2016). Therefore, basic differences in energy partitioning offers a plausible explanation to differences in growth performance between BT and BI steers in the ­present study.

### Carcass characteristics

A concern with introducing BI genetics into the US beef herd in greater proportions is the resulting effects this transition may have on carcass quality (Huffman et al., 1990; Reichhardt et al., 2023). In the US, beef producers are typically paid on a grid system, resulting in YG and QG being pivotal (Schroeder et al. 2009). Previous research has recommended that when steers have at least 50% BI-influence, they will be unacceptable to the cattle feeder if quality grade is used to determine price at harvest (Boyles, 1985). Moreover, Butler et al. (1956b) observed that as diet quality increased, growth performance and quality grades of BT steers increased; but diet quality has little effect on growth performance and quality grades of BT × BI steers. Furthermore, despite Brahman-Angus steers having HCW an average 36.5 kg less than the present study, Boyles and Riley (1991) observed only 10% of Brahman-Angus steers graded USDA Choice, whereas 90% of Angus steers graded USDA Choice when cattle of similar age were evaluated during a 184 d finishing period, similar to the present study. Observations reported by Boyles and Riley (1991) are consistent with the present study where over 90% of BT steers and approximately 29% of BI steers graded USDA Choice. However, it is well known that BT crossbred or purebred steers are expected to exhibit superior meat quality attributes when compared to purebred BI steers (Butler et al., 1956b; Huffman et al., 1990; Goulart et al., 2020). Observations regarding differences in QG and associated marbling score between BT and BI steers may be due to greater selection pressure in BT cattle for economically relevant traits such as superior meat quality (Zayas and Mateescu, 2025). Therefore, in the present study it is reasonable to postulate that even if BI steers were finished to a common final body weight or backfat thickness as BT steers, they would still have less intramuscular fat.

Differences between cattle sub-species regarding intramuscular fat deposition were further confirmed by Reichhardt et al. (2023), stating that marbling score was influenced by cattle breed with BT exhibiting greater marbling scores than BI × BT crosses. In the present study, BT steers had greater marbling scores resulting in all BT carcasses grading USDA Choice or greater, whereas the majority of BI carcasses graded Select. However, the effect of GPT on marbling score differed by cattle sub-species, with the use of GPT resulting in a greater decrease in marbling score for BT steers compared to no observed difference for BI steers. When evaluating Brahman steers that received an anabolic implant, Smith et al. (2007) observed an average marbling score of approximately 390, corresponding to the Select USDA quality grade. This observation is consistent with observations of the present study, where BI steers that were receiving GPT had an average marbling score of 380, corresponding to the Select USDA quality grade. Beyond genetic selection and potential predisposition, differences in energetic efficiency previously discussed regarding growth ­performance may also be a factor influencing muscle versus fat deposition from a carcass standpoint.

In addition to BI carcasses generally having less intramuscular fat, the same has been observed regarding subcutaneous fat deposition. Boyles and Riley (1991) reported that when comparing steers of similar age and FBW (approximately 507 kg) harvested at 184 d on feed, Brahman-Angus steers had 0.5 cm less ­subcutaneous backfat at the 12th rib. Moreover, Boyles and Riley (1991) reported that the LMA of Angus steers was approximately 5 cm^2^ less than BI steers. These observations are consistent with the present study, where BT had approximately 0.3 cm greater FT and 6 cm^2^ smaller LMA, corresponding to greater calculated YG. In the present study, BI steers produced a greater proportion of YG 1 (16% vs. 0%) and YG 2 (51% vs. 7%) carcasses, whereas BT steers produced more YG 3 (60% vs. 27%) and YG 4 (34% vs. 2%) carcasses. Differences in YG can logically be explained by differences in HCW, LMA, and FT between BT and BI steers. For BI steers, lesser calculated YG would be expected due to lighter HCW, less FT, and larger LMA; whereas, for BT steers greater calculated YG would be expected due to heavier HCW, greater FT, and smaller LMA. Boyles and Riley (1991) documented that BT steers had an average YG of 4.0 ± 0.3, while BI steers had an average YG of 3.0 ± 0.3, indicating nearly a fully yield grade difference between BT and BI steers finished at similar FBW and fed for 184 d in cold climatic conditions. These results are similar to the present study, where BT steers had an average calculated YG of approximately 3.5 while BI steers had an average calculated YG of approximately 2.7, demonstrating a similar difference between YG of BT and BI steers as reported by Boyles and Riley (1991). However, in the present study, BT steers were approximately 60 kg heavier after the 180 d feeding period, which may have contributed to some degree to differences in observed carcass characteristics. In the present study, steers were fed to a common number of days on feed rather than to a common compositional endpoint, consistent with approaches reported in the literature. Although, this decision was also influenced by funding constraints and resource availability.

Furthermore, LMA differed with the use of GPT, with GPT+ steers having 6 cm greater LMA than GPT− steers. Due to anabolic implants and beta-adrenergic agonists altering post-absorptive metabolism which influences the deposition of muscle protein (NASEM, 2016), this response was anticipated. In the present study, observations for HCW and LMA are consistent with preceding research investigating the effect of GPT use on carcass characteristics. Bryant et al. (2010) observed that steers receiving an anabolic implant exhibited greater HCW when compared to non-implanted steers, and Bryant et al. (2020) reported that cattle receiving ractopamine hydrochloride exhibited greater HCW than non-supplemented cattle. Additionally, these experiments observed increased LMA when anabolic implants and beta-adrenergic agonists were utilized (Bryant et al., 2010; 2020).

The DP of cattle is an important and economically relevant factor (Butler et al., 1956b). In previous comparative studies, reports vary regarding the outcome of DP between cattle sub-species (BT vs. BI and BI × BT). Some reports suggest that BT cattle have shown an advantage in DP (Adams et al., 1982), while others suggest BI cattle have shown an advantage in DP (Black et al., 1934; Butler et al., 1956a, 1956b), and some report no difference between cattle sub-species (Reichhardt et al., 2023). Black et al. (1934) reported that BI × BT steers had DP between 2 and 4% greater than BT steers. Lunt et al. (1985) reported a numeric difference in DP between Angus (62.8%) and Brahman steers (64.0%). In the present study, observations were consistent with those of Black et al. (1934) and Lunt et al. (1985); BI steers had a DP approximately 4% greater than BT steers, with an average DP of approximately 64% and 62% for BI and BT steers, respectively. Differences in digestive tract size and hide thickness between BI and BT steers offer plausible explanations for differences in DP between sub-species, as BI steers have been observed to have smaller digestive tracts relative to BT steers (Black et al., 1934). Furthermore, DP may not only be influenced by cattle breed, diet, and gut fill, but also by animal age in terms of proximity to mature BW (Coyne et al., 2019). Lunstra and Cundiff (2003) reported that Angus cattle mature at a faster rate than Brahman cattle. Therefore, in the present study, BT steers may have been harvested closer to mature body weight than BI steers. In general, as cattle age and reach mature body weight DP decreases due to the growth of non-carcass components which could logically influence differences in the DP of BT and BI steers in the present study.

### Gas flux

With increased pressure on ruminant livestock industries to decrease CH_4_ emissions, there is a need for continual review of available data to guide future research, policy, and mitigation strategy adoption (Beauchemin et al., 2022). In an assessment of global ruminant CH_4_ emissions measurements, it was observed that the majority of cattle studies have assessed cattle of BT origin, with only 12% of cattle assessed being of BI origin (Della Rosa et al., 2022). However, BI cattle represent more than half the global cattle population and are the predominant cattle sub-species in tropical and subtropical climatic regions of the world (Utsunomiya et al. 2019). Additionally, given their global prominence, greater understanding of CH_4_ emissions from BI cattle fed concentrate-based finishing rations could provide valuable insights into emission mitigation potential of sustainable intensification (FAO, 2023). Given the genomic, phenotypic, and behavioral differences between BT and BI cattle, it is logical to postulate that there may be differences in CH_4_ emissions, which is important to elucidate given the reliance on prediction equations used to estimate emissions. In the nexus between pressure to report progress on climate goals and a lack of representative data of cattle of different sub-species, a need exists to collect gas flux measurements that can be used to evaluate and improve GHG emissions inventories and aid in more accurately representing the contribution of CH_4_ at a global and national scale from beef cattle (Della Rosa et al., 2022).

In the present study, CH_4_ emissions differed by sub-species, with BT steers producing approximately 9% more CH_4_ (g/d) than BI steers; while GPT+ tended to emit more CH_4_ (g/d), than GPT−. Although BI GPT− steers had fewer total visits than other treatments, all steers included in the gas flux analysis met the minimum visitation threshold described by Beck et al. (2024). Nonetheless, fewer BI GPT− steers achieved this threshold compared to other treatment groups. To the authors’ knowledge, previous literature provides guidance only for determining minimum visitation thresholds using the AHCS, and methodologies have not yet been established for defining a minimum number of animals required to accurately represent a population. Future research is therefore needed to evaluate the minimum animal sample size necessary to ensure reliable treatment averages when using the AHCS.

In an evaluation of 3 experiments, Vargas et al. (2024) reported that individual CH_4_ emissions ranged from 150 to 171 g CH_4_/d, similar to the present study, where CH_4_ emissions ranged from 145 to 165 g CH_4_/d. However, despite similarities in gas flux measurement methodologies and data management between the present study and Vargas et al. (2024), only BT steers were used in the three experiments reported by Vargas et al. (2024), whereas the present study also utilized BI and BT steers. Many factors influence CH_4_ emissions include such as the level of feed intake, type and quality of carbohydrates in the diet, feed processing, additives (e.g., lipids and ionophore antibiotics), and alterations in the ruminal microflora (e.g., defaunation; Johnson and Johnson, 1995; Hristov et al., 2013; Beauchemin et al., 2022), prompting a need for further investigation in regard to these two cattle sub-species. To the authors’ knowledge, this is the first study to evaluate gas flux in winter conditions of a temperate US climatic region for this combination of cattle sub-species and management strategies. In addition, future comparative CH_4_ evaluations of BI and BT cattle may consider investigation of the rumen microbial community and rate and extent of carbohydrate digestion to isolate factors influencing CH_4_ production differences between cattle sub-species beyond feed intake in order to identify potential interventions and mitigation opportunities.

A complexity that exists in the realm of sustainability science when applied to animal agriculture regarding gas flux measurement is the interpretation of absolute gas production versus gas production yield and intensity (Beauchemin et al., 2022). This complexity is related to the assessment of tradeoffs across the domains of sustainability to consider aspects of food security, economic viability, and social well-being (Place, 2024). Beyond enteric CH_4_ emissions from beef cattle being an environmental sustainability concern, these emissions are also problematic with respect to energy utilization efficiency (Chaokaur et al., 2015). According to Johnson and Johnson (1995), cattle typically emit between 2 and 12% of their gross energy intake (GEI) as eructated CH_4_. National inventories for enteric CH_4_ are calculated in accordance with the Intergovernmental Panel on Climate Change (IPCC) guidelines, where the CH_4_ conversion factor, also known as *Y*_m_, is a critical metric used to assess the potential extent of global warming and to estimate total enteric CH_4_ emissions (Houghton et al., 1996; Steinfeld et al., 2006; Gerber et al., 2013). The IPCC guidelines for a Tier 2 approach sets a default *Y*_m_ of 3 ± 1% for feedlot cattle fed stem-flaked corn in North America, a value generated predominately utilizing BT gas flux measurements (IPCC, 2006). In the present study, *Y*_m_ ranged from 4.3% to 6.1%, with BI steers having approximately 29% greater *Y*_m_ when compared to BT steers. The present study would suggest that when 3 ± 1% Y_m_ is used in accordance with IPCC Tier 2 guidelines to represent emissions from beef cattle fed a concentrate-based diet during the finishing phase of production, emissions would be underestimated for this population of cattle based on observed *Y*_m_. Moreover, the present study observed that when considering MY, BI steers had approximately 19% greater MY than that of BT. When CH_4_ was expressed as the mass of CH_4_ emitted per unit ADG, BT steers emitted less CH_4_, but the impact of managing steers with GTP resulted in a greater magnitude of difference in EI between GPT− and GPT+ for BI steers compared to BT steers. For BI steers, forgoing the use of GPT resulted in 66% greater EI, whereas for BT steers, this difference was 20%.

According to the IPCC (2019) some factors influencing efficiency of CH_4_ emissions include feed digestibility, breed, level of intake, kinetics of feed passage, rumen volume, rumen ­fermentation profile, and/or the inclusion of other rumen microbial modifiers. When specifically considering the observed *Y*_m_ of the present study, differences between sub-species could be due to the influence of level of feed intake on the kinetics of feed passage through the rumen. As observed, BT steers consumed more feed than BI steers, and when level of feed intake increases, passage rate increases at the sacrament of feed digestibility. Therefore, BT steers are still consuming more total feed which is related to absolute (total) CH_4_ emissions, but as passage rate increases less hydrogen is available per unit of intake for CH_4_ production, thus reducing CH_4_ emitted as a proportion of gross energy intake. Additionally, observed MY supports potential differences in passage rate influenced by differences in feed intake between cattle sub-species. Moreover, observed EI is reasonable considering greater growth rates observed for BT steers compared to BI steers, which could result in the dilution of maintenance energy requirements, meaning a greater proportion of feed energy could have been directed towards growth rather than maintenance for BT steers. The concept of dilution of maintenance and the relation to environmental sustainability of the US beef industry is further discussed in Capper and Bauman (2013).

## Conclusion

The purpose of this study was to evaluate the growth performance, carcass characteristics, and gas flux of BT and BI steers managed with and without the use of GPT in winter conditions of a temperate climate. From a growth performance perspective, despite BI steers starting the trial at heavier IBW, BT steers consumed more feed with greater efficiency resulting in faster rates of growth allowing BT steers to finish at heavier FBW when compared to BI steers. Moreover, GPT increased DMI and G:F, resulting in greater ADG, leading to heavier FBW. Additionally, observations demonstrated that the effect of GPT differed by sub-species for DMI, G:F, HCW, and marbling score. Furthermore, BI steers had carcasses with less FT and calculated YG, but BT steers produced greater quality carcasses which was evident by greater marbling score, highlighting a limitation of BI steers in a consumer demand-driven industry. For gas flux, BI steers emitted less absolute CH_4_ than BT steers; however, BT steers demonstrated lower CH_4_ emissions yield and intensity. Observations highlight the ability for GPT to improve feed utilization efficiency, thus reducing GHG ­emissions when expressed per unit of DMI and ADG.

## Abbreviations:

ADG: average daily gain
AHCS: automated head chamber system
BI: *Bos taurus indicus*
BT: *Bos taurus taurus*
BW: body weight
CH_4_: methane
CO_2_: carbon dioxide
DM: dry matter
DMI: dry matter intake
DMI_BW_: dry matter intake as a percentage of body weight
DP: dressing percentage
EI: emissions intensity
FBW: final body weight
FT: backfat thickness
G:F: gain to feed ratio
GEI: gross energy intake
GHG: greenhouse gas
GPT: growth-promoting technology
H_2_: hydrogen
HCW: hot carcass weight
IBW: initial body weight
IPCC: Intergovernmental Panel on Climate Change
LMA: longissimus muscle area
MY: methane yield
NE_m_: net energy for maintenance
O_2_: oxygen
P: precipitation
QG: quality grade
RH: relative humidity
SBW: shrunk body weight
SR: solar radiation
T_a_: ambient temperature
TMR: total mixed ration
US: United States
USDA: United States Department of Agriculture
WS: wind speed
YG: yield grade
Y_m_: yield of methane
